# Sex-Dependent Metabolic Effects in Diet-Induced Obese Rats following Intermittent Fasting Compared with Continuous Food Restriction

**DOI:** 10.3390/nu16071009

**Published:** 2024-03-29

**Authors:** Laia Bosch de Basea, Marina Boguñà, Alicia Sánchez, Montserrat Esteve, Mar Grasa, Maria del Mar Romero

**Affiliations:** 1 Department of Biochemistry and Molecular Biomedicine, Faculty of Biology, University of Barcelona, 08028 Barcelona, Spain; laiaboschd@gmail.com (L.B.d.B.); marinabp8@gmail.com (M.B.); aliciasg1997@gmail.com (A.S.); 2 CIBER Obesity and Nutrition, Institute of Health Carlos III, 08028 Madrid, Spain; 3 Institute of Biomedicine of the University of Barcelona, 08028 Barcelona, Spain; 4 Department of Biochemistry and Physiology, Faculty of Pharmacy, University of Barcelona, 02028 Barcelona, Spain

**Keywords:** caloric restriction, intermittent fasting, cafeteria diet, insulin resistance

## Abstract

Recently, intermittent fasting has gained relevance as a strategy to lose weight and improve health as an alternative to continuous caloric restriction. However, the metabolic impact and the sex-related differences are not fully understood. The study aimed to compare the response to a continuous or intermittent caloric restriction in male and female rats following a previous induction of obesity through a cafeteria diet by assessing changes in body weight, energy intake, metabolic parameters, and gene expression in liver hepatic and adipose tissue. The continuous restriction reduced the energy available by 30% and the intermittent restriction consisted of a 75% energy reduction on two non-consecutive days per week. The interventions reduced body weight and body fat in both sexes, but the loss of WAT in females was more marked in both models of caloric restriction, continuous and intermittent. Both caloric restrictions improved insulin sensitivity, but more markedly in females, which showed a more pronounced decrease in HOMA-IR score and an upregulation of hepatic IRS2 and Sirt1 gene expression that was not observed in males. These findings suggest the fact that females are more sensitive than males to reduced caloric content in the diet.

## 1. Introduction

The development of obesity-associated metabolic disorders such as insulin resistance and dyslipidemia increase the risk of developing type 2 diabetes mellitus and cardiovascular disease [1]. Evidence suggests that modest body weight loss is among the most important factors for improving glucose and lipid homeostasis [2]. One of the most common current treatments to tackle obesity is caloric restriction (CR). However, low-calorie diets (LCD) maintained over time often result in low adherence and promote physiological changes that reduce energy expenditure and thus prevent further weight loss [3]. In recent years, intermittent fasting (IF) variants have received considerable interest as an alternative approach to losing body weight and improving metabolic health [4]. IF consists of alternating periods of fasting with little or no energy intake with periods of unrestricted feeding [5]. Unlike CR, the molecular regulation involved in the benefits of IF is poorly understood. It has been proposed that abrupt changes in energy status led to changes in fuel utilization that persist during the next refeeding phase. Thus, the repeated periods of mobilization and recovery of energy substrates triggered by IF may favor the reduction of ectopic fat accumulation and promote insulin sensitivity [6]. 

The impact of IF depends on the age, sex, and nutritional and health status of the individual, as well as the selected protocol, its duration, and the proper adherence to the food intake schedule [6,7]. Different approaches have been used for IF interventions: alternate-day fasting [8], the 5:2 diet, in which severe energy restriction is limited to two days a week [9], and daily time-restricted feeding in which food intake is limited to a window of 8–10 h per day [10], usually by extending the daily overnight fasting period. Most rodent studies using IF protocols employ alternate-day total fasting of 24 h; however, the sustainability of this model is questionable in humans as it appears to be one of the most extreme dietary interventions [11]. 

Time-restricted feeding combined with an obesogenic diet for three months avoided fatty liver accrual and improved glucose regulation in mice of both sexes, showing the potential of IF as a preventive of metabolic disturbances approach [12]. In another recent study with a therapeutic strategy, previously fattened male mice with a high-fat diet (HFD) subjected to an alternate-day fasting schedule for 10 weeks lost weight and improved glucose utilization but IF failed to reverse impaired cognitive performance [13]. Interestingly, in such experiments obesogenic diets that have previously fattened the animals are maintained during the IF period. In the therapeutic approach to human obesity treatment, the focus is often on changing not only the caloric intake of the diet but also its composition to a healthier one.

The main drawback of IF studies in humans is that they are inconclusive about how much of the observed benefit is due to the weight loss elicited by total energy restriction (if any) and how much is due to the pattern of intake. Most clinical studies do not allow us to distinguish exactly the metabolic differences triggered by continuous and intermittent energy restriction due to the difficulty in monitoring compliance with the experimental pattern [7]. Therefore, controlled animal studies are still needed to establish the mechanisms and differences between the two strategies. Furthermore, although males and females respond differently to food abundance and scarcity, most rodent studies on intermittent energy restriction only consider the male sex [13,14,15]

This study attempts, on the one hand, to determine whether intermittent energy restriction activates different metabolic mechanisms to those of continuous energy restriction, and on the other hand, to establish sex-specific differences that could contribute to improving dietary treatments to be more effective and personalized. 

These objectives are intended to be examined through a therapeutic procedure in a well-controlled animal model of diet-induced obese male and female rats. To achieve these objectives, rats had free access to a cafeteria diet for 12 weeks, to induce hedonic feeding and hyperphagia that increase body fat. Then, the cafeteria diet was switched to a standard chow under continuous or intermittent restriction for the next 3 weeks.

## 2. Materials and Methods

### 2.1. Animals and Experimental Design

Male and female Wistar rats (Janvier, Le Genest-Saint Isle, France), 5 weeks old, were used. The animals were kept in pairs (fattening period) or individually (intervention period) in transparent wall cages, enabling them to see each other, with wood shards as bedding material and a piece of paper, at 21–22 °C, and 50–60% relative humidity; lights were on from 08:00 to 20:00. All animal handling procedures were approved by the Ethics and Animal Care Committee of the University of Barcelona, following the standards and procedures established by the European, Spanish, and Catalan administrations.

**Figure 1:**
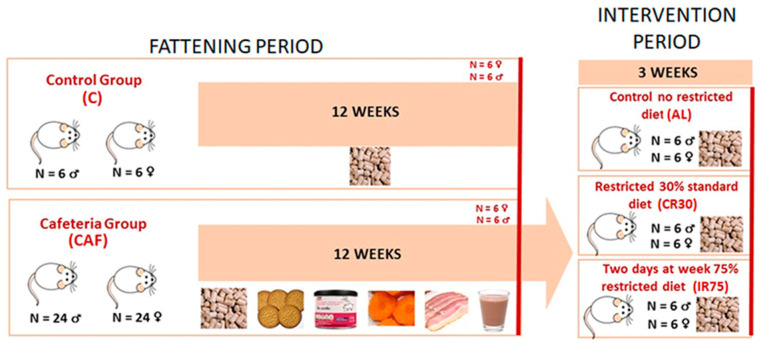
**Experimental protocol scheme**. The cafeteria diet was composed of biscuits, bacon, carrots, pâté, and milk mixed with 22.5% white sugar and 15% cacao. For the explanation, see the text.

The experiment was divided into two time periods: a fattening period with free access to a cafeteria diet and an intervention period in which the rats were fed standard chow ad libitum and under continuous or intermittent restriction (Figure 1). During the fattening period, 6 rats were fed a standard chow diet (#2014, Teklad Diets, Madison, WI, USA) (C group) and 24 rats were fed a cafeteria diet (CAF group) for each sex. After 12 weeks, 6 rats from groups C and CAF were sacrificed to control the changes due to the cafeteria diet and to refer to the effects of further interventions. The remaining cafeteria rats were maintained for 1 week exclusively on a standard chow diet ad libitum to allow habituation to the new diet (stabilization period). Then, during the intervention period, the rats were divided into three experimental groups: a first group was fed ad libitum (AL group), a second group was subjected to a 30% daily energy restriction (continuous restriction or CR30 group), and a third group was subjected to a 75% energy restriction two non-consecutive days per week (intermittent restriction or IR75 group).

The standard chow diet contained 20% of digestible energy derived from protein, 13% from lipids, and 67% from carbohydrates. The cafeteria diet consisted of an oversupply of standard chow pellets, plain cookies spread with liver pâté, bacon, carrot, and milk containing 22.5 g/L sucrose and 10 g/L soluble sweetened cocoa [16,17]. The energy distribution of the cafeteria diet offered was 11% proteins, 55% carbohydrates, and 34% lipids. All components of the cafeteria diet were kept fresh and renewed daily. In all cases, food was supplied in the afternoon to avoid disturbing the circadian rhythm of the rats. During the fattening period, the food consumption of 6 random cages and the body weight of all rats were recorded once a week.

During the stabilization period, food intake and body weight were recorded every day. The average of the last four days food intake was taken as a reference to calculate the restriction to be applied for each rat belonging to the CR30 and IR75 groups. During the intervention period, food and body weight were recorded daily.

Rats were sacrificed between 11:00 am and 1:00 pm, after at least 3 h since food was removed from the cages. In the case of IR75, rats were sacrificed two days after the last fasting day. Rats were killed under isoflurane anesthesia, by exsanguination from the exposed cava. Serum was obtained, and different tissues were extracted and stored at −80 °C until processed.

### 2.2. Estimation of Energy Efficiency and Adiposity Index

Energy efficiency (g/MJ) was calculated for each rat as the mean change in body weight per day in grams during each period (fattening or intervention) divided by the mean energy intake in the same period. This provides a measure of grams of weight gained for each MJ ingested. 

The adiposity index for each rat was calculated from the sum of the weight of the main white adipose tissue depots (perigonadal, retroperitoneal, mesenteric, and subcutaneous inguinal) and was expressed as a percentage of body weight.

### 2.3. Serum Metabolites, Liver Lipid and Glycogen Determinations

Blood serum was used for the measurement of glucose (Biosystems, Barcelona, Spain), non-esterified fatty acids (Wako Chemicals, Neuss, Germany), total triacylglycerols (Biosystems, Barcelona, Spain), lactate (Spinreact, Sant Esteve d’en Bas, Girona, Spain), glycerol (Sigma-Aldrich, St. Louis, MO, USA), and insulin (Ultra Sensitive Rat Insulin ELISA Kit, Crystal Chem’s, Zaandam, The Netherlands). Liver lipids were extracted with trichloromethane: methanol (2:1), dried and weighed [18]. Liver glycogen was quantified as glycosyl residues using anthrone reagent [19]. 

### 2.4. Gene Expression Analysis 

Total tissue RNA from liver and perigonadal adipose tissue was extracted using the Tripure reagent (Roche Applied Science, Indianapolis, IN, USA). RNA content was quantified in an ND-1000 spectrophotometer (NanoDrop Technologies, Wilmington, DE, USA). RNA samples were reverse transcribed using oligo-dT primers (Gene Link, Westchester, NY, USA) and the MMLV reverse transcriptase (Promega, Madison, WI, USA) system. Real-time PCR amplification was carried out using 10 μL amplification mixtures containing Power SYBR Green PCR Master Mix (Applied Biosystems, Foster City, CA, USA), 4 ng of reverse-transcribed RNA, and 150 nM of corresponding primers. Reactions were run on an ABI PRISM 7900 HT detection system (Applied Biosystems). *Arbp* was the charge control gene. The genes analyzed and the primers used were as follows: ATP Citrate Liasa (Acly) forward: 5′-TGTGCTGGGAAGGAGTATGG-3′ reverse: 5′-GCTGCTGGCTCGGTTACAT-3′; Acetyl-coenzyme A carboxylase alpha (Acaca) forward: 5′-TCTACATCCGCTTGGCTGAG-3′ reverse: 5′-ACTCCTCCCGCTCCTTCAAC-3′; Fatty acid synthase (Fasn) forward: 5′-CCCGTTGGAGGTGTCTTCA-3′ reverse: 5′-AAGGTTCAGGGTGCCATTGT-3′; Glycerol-3-phosphate acyltransferase, mitochondrial (Gpam) forward: 5′-GGTGAGGAGCAGCGTGATT-3′ reverse: 5′-GTGGACAAAGATGGCAGCAG-3′; Carnitine Palmitoyltransferase 1a (Cpt1a) forward: 5′-CCGCTCATGGTCAACAGCA-3′ reverse: 5′-CAGCAGTATGGCGTGGATGG-3′; Peroxisome proliferator-activated receptor alpha (Ppara) forward: 5′-TTCAATGCCCTCGAACTGGA-3′ reverse: 5′-GCACAATCCCCTCCTGCAAC-3′; Lactate dehydrogenase a (Ldha) forward: 5′-AAAGGCTGGGAGTTCATCCA-3′ reverse: 5′-CGGCGACATTCACACCACT-3′; Monocarboxylate transporter 1(Mct1) forward: 5′-CCCAGAGGTTCTCCAGTGCT-3′ reverse: 5′-ACGCCACAAGCCCAGTATGT-3′; Sirtuin 1 (Sirt1) forward: 5′-AGAACCACCAAAGCGGAAA-3′ reverse: 5′-TCCCACAGGAAACAGAAACC-3′; Insulin receptor substrate 2 (Irs2) forward: 5′-CATCCACATCCCCAGGACAG-3′ reverse: 5′-CCAGGACAGCCAATCAAAGC-3′; Pyruvate dehydrogenase kinase 4 (Pdk4) forward: 5′-CTGCTCCAACGCCTGTGAT-3′ reverse: 5′-GCATCTGTCCCATAGCCTGA-3′; Phosphoenolpyruvate carboxykinase 1 (Pck1) forward: 5′-CGGGTGGAAAGTTGAATGTG-3′ reverse: 5′-AATGGCGTTCGGATTTGTCT-3′; Solute carrier family 2 member 4 (Scl2a4) forward: 5′-TTCCAGTATGTTGCGGATGC-3′ reverse: 5′-GTGAAGATGAAGAAGCCAAGCA-3; Lipoprotein Lipase (Lpl) forward: 5′-TGGCGTGGCAGGAAGTCT-3′ reverse: 5′-CCGCATCATCAGGAGAAAGG-3′; Hormone-sensitive lipase (Hsl) forward: 5′-TCCTCTGCTTCTCCCTCTCG-3′ reverse: 5′-ATGGTCCTCCGTCTCTGTCC-3′; Aquaporin 7 (Aqp7) forward: 5′-ACAGGTCCCAAATCCACTGC-3′ reverse: 5′-CCGTGATGGCGAAGATACAC-3′; Acidic ribosomal phosphoprotein P0 (Arbp) forward: 5′-GAGCCAGCGAAGCCACACT-3′ reverse: 5′-GATCAGCCCGAAGGAGAAGG-3′.

### 2.5. Statistical Procedures

The Statgraphics Centurion 18^©^ program was used for statistical analysis (Statgraphics Technologies, Inc., The Plains, VA, USA). Data were verified for normality (ShapiroWilk– test) and homoscedasticity (Levene’s test). Two statistical analyses were carried out using two-way ANOVA (sex and diet factors), one to compare C and CAF groups (fattening period) and the other to compare CAF, AL, CR30, and IR75 groups (intervention period). Within each sex, differences between groups were determined using a one-way ANOVA and a Tuckey post-test. T-tests have been used to assess differences between sexes in macronutrient intake from cafeteria diet during the fattening period.

## 3. Results

### 3.1. Food Intake

**Figure 2:**
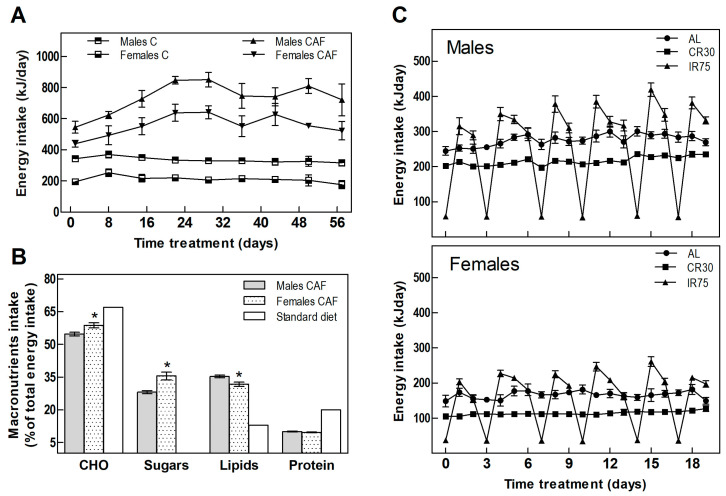
**Energy intake.** (**A**) Total daily energy intake in the fattening period. (**B**) Energy percentage from macronutrients of cafeteria diet consumed by males and females compared to the standard diet. (**C**) Total daily energy intake in males and females through the treatment period. Data are expressed as mean ± SEM. Statistical differences between males and females were assessed by *t*-student, * *p* < 0.001.

Table 1 shows the food intake and body weight of animals throughout the experiment. In the fattening period, CAF groups increased their energy intake compared with the C groups by 119% and 164% in males and females, respectively (Table 1 and Figure 2A). The cafeteria diet offered to the rats was rich in fats and sugars, in contrast to the standard chow diet, which provides less than half the lipids and scarce sugars (Figure 2B). Analysis of the composition of the rats’ self-selected food revealed a different sex-dependent behavior. As expected, rats of both sexes increased lipid and sugar intake; however, males consumed more lipids while females showed more craving for carbohydrates and sugar (Figure 2B).

Switching from the cafeteria diet to the standard chow diet ceased the hyperphagia experienced by rats fed a CAF diet. IR75 rats, subjected to intermittent food restriction two days a week, increased their intake on the days following severe food restriction (Figure 2C), eating globally the same amount as AL rats (Table 1). Thus, the only groups that decreased their overall chow consumption were the continuously restricted CR30 groups (Table 1).

### 3.2. Body Weight

Free access to a cafeteria diet resulted in a significant increase in body weight compared to the C group in both sexes (Table 1). The cafeteria diet promoted a weight gain of 223% in males and 133% in females in 12 weeks, whereas the chow standard diet in C groups increased body weight by 154% and 66% in males and females, respectively. Therefore, the impact of a cafeteria diet on body weight was heavier in females, growing up to 101%, than in males, who achieved 44% more weight than the C group.

After 12 weeks of cafeteria diet, changing to the chow standard diet offered ad libitum (AL groups) decreased the body weight in females by 7.6% and to a lesser extent in males, by 1.3%. These reductions were comparable to those elicited by intermittent restriction (IR75 groups) in 7.7% and 2% in males and females, respectively. Continuous standard chow restriction (CR30 groups) resulted in a deep decrease in body weight in both sexes, up to 12% in males and 15% in females. These reductions in CR30 groups were statistically significant compared to those observed in AL and IR75 groups. 

### 3.3. Energy Efficiency

As shown in Table 1, sexual dimorphism was observed in terms of energy efficiency, with females being less efficient than males in all the experimental groups. Cafeteria diet decreased energy efficiency in both sexes, but in males the reduction, compared with C groups, was higher (60%) than in females (32%). 

Furthermore, all three dietary interventions following the cafeteria diet significantly decreased energy efficiency in both sexes, more markedly in the case of CR30 groups. In these cases, a negative energy efficiency indicated body mass mobilization. 

### 3.4. Fat Depots, Adiposity Index, and Liver Lipid and Glycogen Content

**Figure 3:**
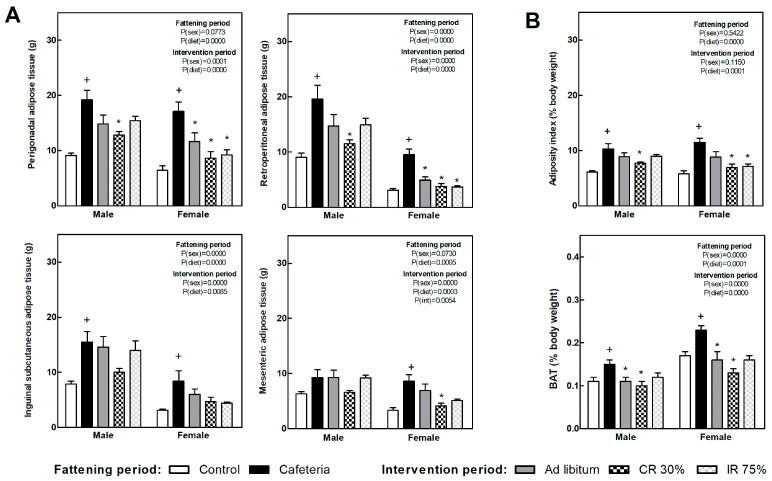
**Adipose tissues’ weight at the end of fattening and intervention periods.** (**A**) Weight of main white adipose tissue (WAT) depots. (**B**) Adipose index, calculated as the sum of four WAT depots expressed as body weight percentage (top graph) and brown adipose tissue (BAT) as a percentage of body weight (bottom graph). Data are expressed as mean ± SEM. Statistical analysis in the fattening and intervention periods was evaluated through two-way ANOVA (sex and diet factors). Differences between C and CAF groups were assessed by *t*-student (+). For the intervention period and each sex, a one-way ANOVA and a Tuckey post-test were performed to identify differences with the CAF group (*). Significant differences were considered when *p* < 0.05. Only significant interactions between factors (P(int)) are shown.

Figure 3A shows several white fat depot weights in each of the experimental groups. Retroperitoneal and perigonadal depot showed the highest expandability in response to the nutrient overplus and were the most responsive to the subsequent dietary intervention. The adiposity index (Figure 3B) was doubled due to the cafeteria diet, while it significantly decreased after continuous food restriction in both sexes, i.e., CR30 groups, and intermittent restriction intervention (IR75) only in females. The brown adipose tissue (BAT), expressed as body weight percentage (Figure 3B), increased in the CAF groups compared to the C groups. All the dietary interventions decreased BAT weight compared to the CAF group in both sexes. 

**Figure 4:**
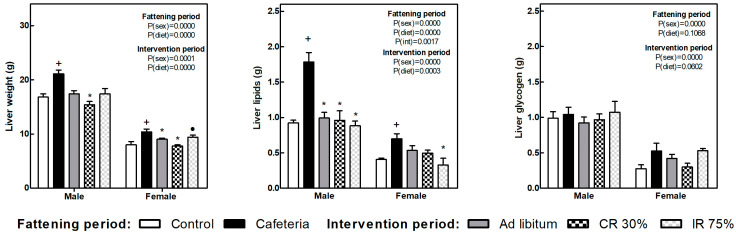
**Liver weight, liver lipids, and liver glycogen at the end of the fattening and intervention periods.** Data are expressed as mean ± SEM. Statistical analysis in the fattening and intervention periods was evaluated through two-way ANOVA (sex and diet factors). Differences between C and CAF groups were assessed by *t*-student (+). For the intervention period and each sex, a one-way ANOVA and a Tuckey post-test were performed to identify differences with the CAF group (*), and between CR30 and IR75 (●). Significant differences were considered when *p* < 0.05. Only significant interactions between factors (P(int)) are shown.

The liver weight increased in both sexes because of the cafeteria diet intake (Figure 4). All the dietary interventions tend to reduce liver weight, and the decrease was significant in the CR30 groups in both sexes and the AL group only in females. Also in females, the decrease in liver weight observed in the IR75 group was less pronounced, leading to differences between CR30 and IR75.

Liver lipid content also increased in response to cafeteria diet in both sexes and decreased in all the dietary interventions, deeply in males. In females, the reduction of liver lipids was more marked in the IR75 group (Figure 4). Glycogen liver content did not change under any condition.

### 3.5. Serum Parameters

Table 2 shows the serum levels of metabolites in male and female rats after the fattening and the intervention period. The cafeteria diet increased insulin and HOMA-IR score in both sexes, but only glucose levels were raised in males. Cafeteria diet also promoted increased triacylglycerol levels in both sexes, while NEFA and glycerol increased only in males.

After the intervention period, glucose levels decreased and were normalized in both males and females, although it was more pronounced in the IR75 group. Serum insulin also decreased overall during the intervention period and, comparing within each sex, the drop in insulin levels was significant in the male CR30 group and the female IR75 group. The HOMA-IR score decreased after the return to the standard chow diet, more deeply in the food-restricted groups, but with differences between sexes. Thus, while the male AL group restored the HOMA-IR score to baseline values, females did not normalize it. Circulating triacylglycerol levels decreased in all dietary interventions in both sexes. In males, the plasma levels of NEFA and glycerol reverted to control group values after the intervention period.

### 3.6. Liver Gene Expression

**Figure 5:**
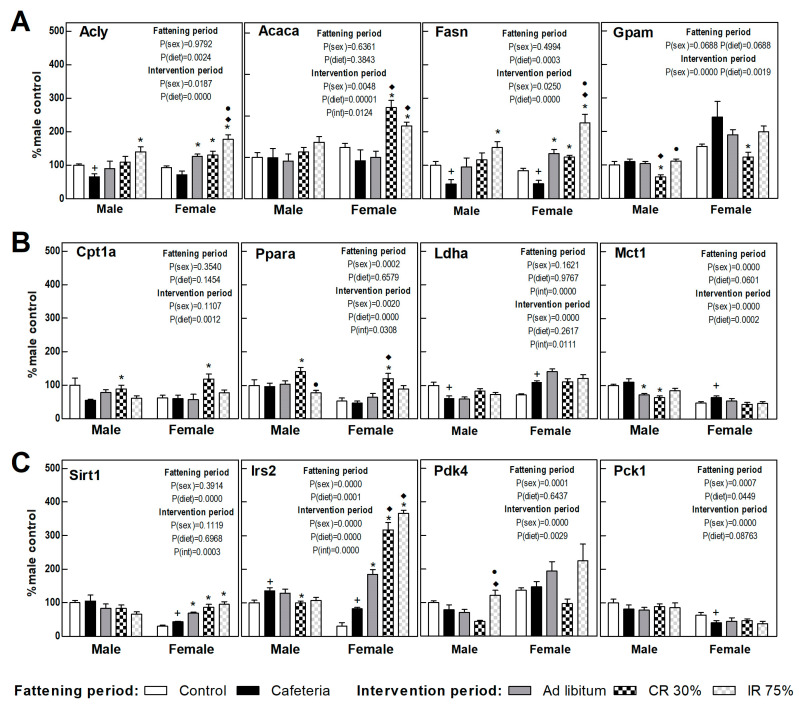
**Gene expression in the liver.** (**A**) Proteins related to lipogenesis and fatty acid esterification to triacylglycerols. (**B**) Proteins related to fatty oxidation and lactate synthesis and transport. (**C**) Proteins related to insulin signaling and glucose metabolism. Data are expressed as mean ± SEM. Statistical analysis in the fattening and intervention periods was evaluated through two-way ANOVA (sex and diet factors). Differences between C and CAF groups were assessed by *t*-student (+). For the intervention period and each sex, a one-way ANOVA and a Tuckey post-test were performed to identify differences with the CAF group (*), the AL group (♦), and between CR30 and IR75 (●). Significant differences were considered when *p* < 0.05. Only significant interactions between factors (P(int)) are shown.

The expression of proteins involved in the liver energy metabolism is shown in Figure 4, calculated as a percentage of those observed in control males. The cafeteria diet promoted a decrease in the expression of genes involved in hepatic de novo lipogenesis (DNL) (Acly and Fasn) (Figure 5A) and an increase in Irs2 gene expression (Figure 5C) in both sexes. The cafeteria diet also produced sex-dependent changes, since only in females did it increase lactate synthesis and transport (Ldha, Mct1 genes) and Sirt1, and it decreased the expression of the rate-limiting gene of gluconeogenesis, Pck1 (Figure 5B,C).

In females, the return to the ad libitum standard diet (AL Group) increased the DNL-gene expression, Sirt1, and Irs2 compared to the CAF group (Figure 5A,C). The only change observed in AL male groups was a decrease in the lactate transporter (Mct1) gene expression compared to the CAF group (Figure 5B). The continuous restriction diet (CR30) promoted in both sexes a decrease in the genes involved in fatty acid esterification (Gpam) and an increase in those involved in lipid oxidation (Cpt1a and Ppara genes) versus the CAF and AL group (Figure 5A,B). In addition, sex differences were observed in CR30 intervention in respect to the cafeteria diet. Only in females were DNL genes, Irs2, and Sirt1 highly expressed (Figure 5A,C). The intermittent restriction groups, IR75, further increased the gene expression of DNL and Pdk4 genes, especially in females, pointing to a metabolic switch to lipogenesis. Continuous caloric restriction (CR30) showed a pattern consistent with increased lipid oxidation that was not observed in intermittent restriction (Figure 5A–C). Globally, all dietary interventions seemed to promote DNL, especially in females and in intermittent fasting restriction. Continuous caloric restriction enhanced lipid oxidation in both sexes. All dietary interventions pointed to an improvement in insulin resistance in females, a fact not observed in males.

### 3.7. Perigonadal Adipose Tissue Gene Expressions

**Figure 6:**
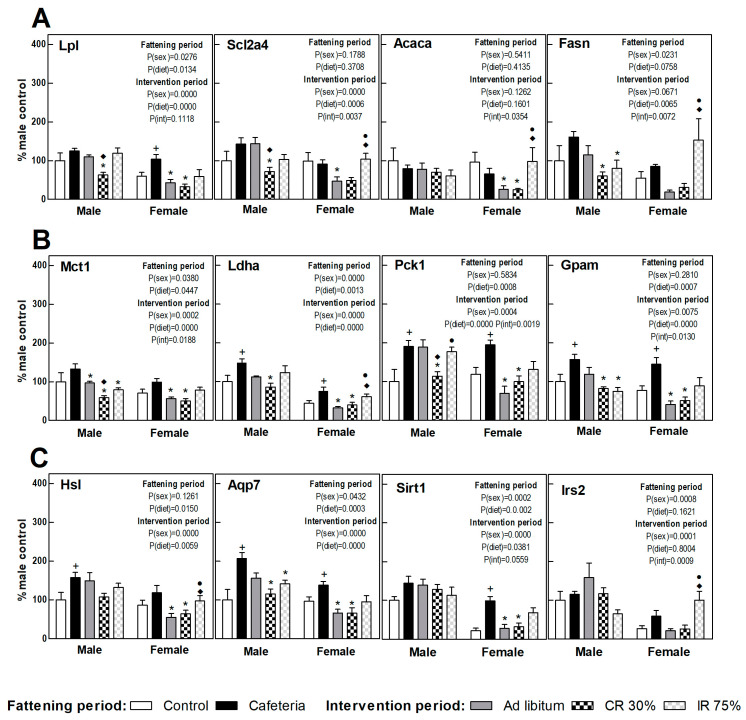
**Gene expression in perigonadal WAT.** (**A**) Proteins related to fatty acids and glucose uptake and lipogenesis. (**B**) Proteins related to lactate transport/production, glycerol-3P synthesis, and fatty acid esterification to triacylglycerols. (**C**) Proteins related to lipolysis, glycerol output, and insulin signaling. Data are expressed as mean ± SEM. Statistical analysis in the fattening and intervention periods was evaluated through two-way ANOVA (sex and diet factors). Differences between C and CAF groups were assessed by *t*-student (+). For the intervention period and each sex, a one-way ANOVA and a Tuckey post-test were performed to identify differences with the CAF group (*), the AL group (♦), and between CR30 and IR75 (●). Significant differences were considered when *p* < 0.05. Only significant interactions between factors (P(int)) are shown.

Figure 6 shows the perigonadal adipose tissue gene expression in the fattening and intervention periods in both sexes, calculated as a percentage of control males. A clearly increased FA uptake (Lpl) and TAG turnover was observed under the cafeteria diet in both sexes (esterification, Gpam gene; lipolysis and glycerol synthesis, Hsl, Ldha, Pck1, and Aqp7 gene) (Figure 6). Sirt1 and Irs2 gene expression increased deeply in females under the cafeteria diet (Figure 6C).

The return to the ad libitum standard diet (AL groups) caused a different response in male and female rats. Thus, while in females a decrease was observed in the gene expression of practically all the genes studied compared to the CAF group, in males only the expression of genes involved in lactate synthesis and transport (Ldha and Mct1) was significantly decreased (Figure 5). Continuous restriction intervention (CR30 groups) also showed a sex-related response, since the CR30 group in females showed a pattern of gene expression very similar to that observed in the AL group. However, in males, the response of the CR30 group was more marked than in the AL group, decreasing significantly the expression of genes involved in nutrient uptake (Scl2a4, Lpl), glyceroneogenesis (Pck1), and lactate transport (Mct1) compared to the AL group. In females, the general pattern of expression in intermittent fasting (IR75 group) was similar in respect to the CAF group, whereas in males there was a decreased TAG synthesis and turnover (Fasn, Gpam, Mct1, and Aqp7 genes). These results indicate that the perigonadal WAT of females would be more sensitive to the shift to a standard diet (less lipids and sugars) than to an energy restriction, whereas the WAT of males would require a severe reduction of dietary energy to respond.

## 4. Discussion

IF is a weight loss dietary intervention that has recently gained prominence over continuous CR [4]. However, its metabolic impact is not fully understood [20], nor whether it is similar in males and females because studies in experimental animals are mostly carried out in males [13,14]. In the present study, we set out to compare the response to a dietary intervention with continuous or intermittent caloric restriction in rats of both sexes, to evaluate changes in energy intake, weight loss, metabolic parameters, and gene expression of metabolic enzymes in liver and adipose tissue. A pre-fattening period with a human-like palatable diet (CAF) to induce obesity was followed by an intervention period (AL, CR, or IR) in which animals were fed a standard diet, thus better simulating a human dietary intervention. Unlike most intermittent fasting studies, in which during the intervention period rodents are still fed the diet used for fattening, this study combined both diet composition changes and feeding pattern interventions [12,13,14].

The cafeteria diet, as expected, led to a larger amount of intake and body weight increases [21], resulting in a rise in the weight of the different WAT depots and BAT, as well as in the hepatic lipid content in both males and females [22]. In addition, the cafeteria diet promoted lower energy efficiency to counteract the higher caloric intake and to increase energy expenditure by a larger BAT [21,23,24]. Interestingly, females showed a lower energetic efficiency than males, in agreement with a previous study [16]. Furthermore, a higher preference for sugars has been found in females and fats in males under free access to different foods during the cafeteria diet. In humans, females show more cravings for sweet foods and males for protein foods, a behavior that combines biology and cultural patterns [25]. In rats, where only biology is taken into account, few data are available. The main data have been obtained from conditioned taste aversion studies, showing again in females a higher preference for the intake of a sweet solution compared with males [25]. A recent study using a cafeteria diet failed to show differences in food choice between sexes, but only short-term periods of 8 days were assessed [26].

The expression pattern of genes involved in lipid metabolism observed in liver and adipose tissue due to the cafeteria diet in both sexes was consistent with a decrease in DNL and an increased deposition of dietary lipids [27,28,29]. Only in the liver of female rats did glucose metabolism show a switch towards lactate production and lipid oxidation, indicated by an increase in Ldha, Mct1, and Sirt1 expression. Although the cafeteria diet increased insulin resistance in both sexes, with an increase in HOMA-IR score, female rats maintained glucose levels, while glucose raised significantly in males, consistent with the protective role of estrogens on insulin action [30]. Impaired circulating levels of TAG and NEFA were also observed due to the cafeteria diet only in males, in agreement with a previous study [16].

The increase in perigonadal WAT expression of Pck1, Gpam, and Aqp7 genes agreed with an increase in glyceroneogenesis to support increased TAG turnover, also in line with the increase of Hsl expression. Although increased Pck1 and glyceroneogenesis in WAT have been generally associated with fasting [31], other studies show that Pck1 is primarily responsible for the generation of glycerol-3P for fatty acid esterification in the WAT [32]. Even though not all studies are in the same direction, it has been described that increased fatty acid re-esterification by Pck1 overexpression at the adipose tissue leads to obesity [33].

Returning to a standard chow pellet after a period of cafeteria diet elicited a decrease in intake, probably due to the loss of the hedonic component of eating [34,35], which was reflected in a drop in body weight and accordingly in a negative energy efficiency. A different response in energetic efficiency was found between males and females, so females showed a more pronounced decrease. In addition, females also displayed a decrease in perigonadal and retroperitoneal WAT depots consistent with a gene expression profile of fatty acid depletion and limited glucose uptake and DNL. The increasing DNL in the liver of females was supported by the increased hepatic gene expression of Acly and Fasn in response to the lower amount of fat in the standard pellet diet, as previously found [27,28].

As expected, continuous restriction (CR30) elicited a decrease in body weight and a negative energy efficiency and adiposity index in both males and females [36]. Perigonadal WAT incorporated less glucose and fatty acids and decreased TAG turnover according to the low insulin signaling [31]. In addition, Pck1 expression was diminished, a response in accordance with a reduction of WAT size and lipid content [37]. Interestingly, in the liver, an increased gene expression of lipolytic enzymes (Ppara and Cpt1) was observed in both sexes, but only in females did it coexist with an elevated DNL (Agly and Fasn). CR also promoted an improvement in insulin resistance, with a significant reduction in HOMA-IR score. The effects of CR have been extensively studied in terms of longevity, describing a species- and even a strain-dependent sexual dimorphism in rodents [38,39]. The mechanisms by which CR promotes longevity are the same as those that promote weight loss and increased insulin sensitivity, i.e., improved health [40,41,42]. In this regulation, sirtuins and AMPK play a relevant role as nutrient sensors that increase insulin sensitivity and lipid oxidation [42,43,44,45]. Accordingly, the CR30 group showed increased hepatic expression of Sirt1 and Irs2, but only in females, with no change in males. Furthermore, it has been described that GH and IGF1 also play a key role in the response to CR. These hormones display a marked sexual dimorphism that could help to understand the different response in males and females to CR [43].

A variety of scheduling approaches have been studied in order to ascertain IF consequences in energy metabolism [5,11]. Here, we applied a 75% energy restriction on two non-consecutive days per week (IR75 groups). Despite rats of both sexes showing the same body weight and food intake as ad libitum rats, only female mice subjected to IR75 achieved a reduction in fat mass comparable to that observed in the continuous restriction group (CR30). In addition, females exhibited an increased gene expression of DNL-related proteins in both liver and perigonadal WAT compared to CR30 treatment. These differences in DNL-related expressions between CR30 and IR75 could be partially attributed to the fact that the IR75 mice group was sacrificed two days after restriction when they were eating ad libitum. In addition, increased adipocyte DNL seems to be a specific female strategy to cope with metabolic insults in order to maintain insulin sensitivity [46], a metabolic response that we have observed in the female group under intermittent fasting.

IR75 intervention showed an increase in Pdk4 hepatic gene expression in males and females in concordance with the reduced liver lipid content and the improved HOMA-IR score.

The term metabolic flexibility refers to the ability of the organism to switch rapidly between glucose and fatty acid oxidation during the transition between feeding and fasting states [47]. Some pathological conditions, such as diabetes, non-alcoholic fatty liver disease, and consumption of high-calorie diets, lead to a loss of metabolic flexibility, whereas calorie restriction and IF increase it [43,47].

In our study, switching to a balanced diet and spontaneous reduction of energy intake was able to restore hepatic lipid content and HOMA-IR score in both sexes. The decrease in WAT weight, together with a lower expression of genes involved in DNL and TAG turnover, support a deeper sensitivity and metabolic flexibility in WAT of females, even when facing only the change from a cafeteria diet to a standard diet. The loss of WAT in females is more marked in both models of caloric restriction, continuous and intermittent, regardless of changes in body weight, whereas in males only continuous energy restriction was able to reduce WAT stores.

## 5. Conclusions

After a period of fattening on a high-fat, high-sugar diet (CAF), the transition to a balanced diet feeding ad libitum (AL) or intermittent restriction (IR75) promoted a similar overall intake, weight loss, and energy efficiency, while continuous caloric restriction (CR30) resulted in greater weight loss but also much lower energy efficiency. Considering white adipose tissue, IF elicited the same loss as CR in females but not in males, where CR was the more effective intervention. The return to the standard diet normalized the HOMA-IR score, and both caloric restrictions further improved insulin sensitivity. The results presented here point to the fact that females are more sensitive to the quality and quantity of the caloric content of the intake. Sex is a mandatory factor to consider in dietary interventions to improve metabolic disturbances associated with diet-induced obesity.

## Figures and Tables

**Table 1:** Food intake and body weight of male and female rats during the fattening and the intervention period.

	Fattening Period (12 Weeks)				Intervention Period (3 Weeks)											
Parameter	Males		Females		*p*-Value (ANOVA)			Males			Females			*p*-Value (ANOVA)		
	Control	Cafeteria	Control	Cafeteria	Sex	Diet	Int	AL	CR30	IR75	AL	CR30	IR75	Sex	Diet	Int
Type of restriction	Unrestricted		Unrestricted					Unrestricted	Daily	Twice a week	Unrestricted	Daily	Twice a week			
Food type	Standard	Cafeteria	Standard	Cafeteria				Standard	Standard	Standard	Standard	Standard	Standard			
Food available	-	-	-	-				-	70%	25%	-	70%	25%			
Initial body weight (g)	192 ± 2		161 ± 2					623 ± 75	602 ± 30	608 ± 50	356 ± 30	356 ± 32	342 ± 13			
Final body weight (g)	526 ± 7	608 ± 11 ^+^	283 ± 10	368 ± 7 ^+^	0.0000	0.0000	0.8911	615 ± 73	531 ± 25 *	595 ± 54	329 ± 23 *	304 ± 31 *	316 ± 8 *	0.0000	0.0001	0.1999
BW increase (%)	154 ± 5	223 ± 5 ^+^	66 ± 3	133 ± 4 ^+^	0.0000	0.0000	0.8787	−1.3 ± 0.5 *	−11.7 ± 1.7 *^♦^	−2.0 ± 2.2 *^●^	−7.6 ± 2.3 *	−14.7 ± 2.5 *^♦^	−7.7 ± 1.5 *^●^	0.0000	0.0000	0.0000
Food consumption (KJ/day)	335 ± 6	735 ± 15 ^+^	212 ± 7	558 ± 13 ^+^	0.0000	0.0000	0.0000	261 ± 8 *	176 ± 5 *^♦^	246 ± 10 *^●^	156 ± 4 *	110 ± 5 *^♦^	149 ± 4 *^●^	0.0000	0.0000	0.0000
Energy efficiency (g/MJ)	15.2 ± 0.7	9.1 ± 0.2 ^+^	8.8 ± 0.4	6.0 ± 0.2 ^+^	0.0000	0.0000	0.0001	−1.5 ± 0.3 *	−20.3 ± 1.9 *^♦^	−2.7 ± 1.4 *^●^	−9.0 ± 1.6 *	−24.1 ± 2.5 *^♦^	−7.1 ± 1.6 *^●^	0.0000	0.0000	0.1645

Data are expressed as mean ± SEM. Statistical analysis in the fattening and intervention periods was evaluated through two-way ANOVA (sex and diet factors). Differences between C and CAF groups were assessed by *t*-student (^+^). For the intervention period and each sex, a one-way ANOVA and a Tuckey post-test were performed to identify differences with the CAF group (*), the AL group (♦), and between CR30 and IR75 (●). Significant differences were considered when *p* < 0.05. Interactions between factors Int are shown.

**Table 2:** Serum parameters of male and female rats during the fattening and the intervention period.

		Fattening Period (12 Weeks)							Intervention Period (3 Weeks)								
Parameter	Units	Males		Females		*p*-Value (ANOVA)			Males			Females			*p*-Value (ANOVA)		
		Control	Cafeteria	Control	Cafeteria	Sex	Diet	Int	AL	CR30	IR75	AL	CR30	IR75	Sex	Diet	Int
Glucose	mM	5.52 ± 0.14	7.07 ± 0.13 ^+^	6.47 ± 0.14	6.99 ± 0.31	0.4006	0.0356	0.4622	5.99 ± 0.28 *	5.77 ± 0.39 *	4.99 ± 0.06 *^♦^	6.56 ± 0.33	5.44 ± 0.22 *	5.08 ± 0.31 *^♦^	0.7421	0.0000	0.4197
Insulin	pM	289 ± 38	443 ± 60	173 ± 33	321 ±75	0.0389	0.0113	0.9601	304 ± 35	248 ± 37 *	289 ± 37	263 ± 48	149 ± 26	123 ± 29 *	0.0022	0.0008	0.5974
HOMA-IR score		12.6 ± 1.7	20.3 ± 2.7 ^+^	7.6 ± 1.5	15.5 ± 4.0 ^+^	0.0736	0.0079	0.9984	12.3 ± 1.0 *	9.3 ± 1.1 *	9.7 ± 1.3 *	11.5 ± 2.1	5.3 ± 0.8 *	4.2 ± 1.1 *	0.0123	0.0000	0.6806
Triacylglycerol	mM	1.01 ± 0.10	2.74 ± 0.18 ^+^	0.64 ± 0.05	1.10 ± 0.16	0.0000	0.0000	0.0004	1.18 ± 0.18 *	1.13 ± 0.11 *	1.15 ± 0.12 *	0.73 ± 0.14 *	0.47 ± 0.04 *	0.86 ± 0.09 *	0.0000	0.0000	0.0001
NEFA	mM	0.24 ± 0.03	0.36 ± 0.04 ^+^	0.27 ± 0.03	0.23 ± 0.03	0.0428	0.0991	0.0286	0.28 ± 0.05 *	0.23 ± 0.01 *	0.24 ± 0.02 *	0.23 ± 0.03	0.25 ± 0.02	0.23 ± 0.02	0.1763	0.5618	0.1872
Glycerol	mM	0.16 ± 0.02	0.43 ± 0.08 ^+^	0.19 ± 0.05	0.22 ± 0.02	0.1370	0.0031	0.0297	0.17 ± 0.02 *	0.17 ± 0.02 *	0.18 ± 0.01 *	0.17 ± 0.01	0.18 ± 0.03	0.12 ± 0.01 *	0.0051	0.0000	0.0051
Lactate	mM	2.94 ± 0.19	2.98 ± 0.36	1.75 ± 0.11	2.30 ± 0.24	0.0016	0.2608	0.3172	2.51 ± 0.23	2.39 ± 0.37	2.68 ± 0.20	2.05 ± 0.2	1.78 ± 0.09	2.10 ± 0.18	0.0043	0.0340	0.8563

Data are expressed as mean ± SEM. Statistical analysis in the fattening and intervention periods was evaluated through two-way ANOVA (sex and diet factors). Differences between C and CAF groups were assessed by *t*-student (^+^). For the intervention period and each sex, a one-way ANOVA and a Tuckey post-test were performed to identify differences with the CAF group (*), the AL group (♦). Significant differences were considered when *p* < 0.05. NEFA (Non-Esterified Fatty Acids), HOMA-IR score (Homeostatic Model Assessment for Insulin Resistance). Interactions between factor Int are shown.

## Data Availability

Data are contained within the article.
